# Relation of Brain to Cranial Capacity

**Published:** 1915-05

**Authors:** 


**Relation of Brain to Cranial Capacity.** Rudolph, Semaine Med. From examinations of 111 cadavers within 24 hours after death, the following conclusions are drawn: The dura, both in infants and adults, occupies about 3.5% of the cranial space. At birth, the brain occupies 97.5% of the cranial space. By the age of 6, this is reduced to 97%. At puberty, the brain occupies 92.5% of the cranial space, this figure being nearly uniform for both sexes and persisting to middle life. The brain then gradually shrinks so that, in old age, its volume is about 85% of the cranial capacity. The subdural space amounts in adults to about 3%. Pagenstecher, in animal experiments, showed that injection of the subdural space could be made up to 2.9% of the total cranial capacity, before compression symptoms began and that the maximum without producing death from compression, was 6.5%. Rudolph does not think that these percentages apply to human beings.

